# The Effect of Dietary Fat and Sucrose on Cognitive Functioning in Mice Lacking Insulin Signaling in Neuropeptide Y Neurons

**DOI:** 10.3389/fphys.2022.841935

**Published:** 2022-04-26

**Authors:** Caitlin S. Mitchell, Elisabeth K. Goodman, Caitlin R. Tedesco, Kathy Nguyen, Lei Zhang, Herbert Herzog, Denovan P. Begg

**Affiliations:** ^1^ School of Psychology, UNSW Sydney, Sydney, NSW, Australia; ^2^ Garvan Institute of Medical Research, Darlinghurst, NSW, Australia

**Keywords:** hippocampus, spatial cognition, morris water maze, diet, NPY (neuropeptide Y)

## Abstract

Obesogenic diets can produce hippocampal insulin resistance and impairments to hippocampal-dependent cognition. This study investigated the effect of disrupted insulin signaling in Neuropeptide Y (NPY) neurons on diet-induced deficits in hippocampal-dependent memory. Wild-type mice and mice that had a targeted knockout of insulin receptors on NPY cells (IR^lox/lox^;NPY^Cre/+^) were given *ad libitum* access to a high-fat diet (high fat; HF), 10% sucrose solution (high sugar; HS), both high-fat diet and sucrose solution (high fat, high sugar; HFHS), or a normal fat control chow for 12 weeks. Mice were tested in the Morris Water Maze (MWM), a hippocampal-dependent spatial memory task. Glucose homeostasis was assessed via a glucose tolerance test. Independent of genotype, consumption of HF, but not HS, diet increased energy intake, body weight, and plasma leptin, and impaired glucose tolerance. Disrupted insulin signaling in NPY cells and dietary interventions did not significantly affect the ability of mice to learn the location of the platform in the MWM. However, for IR^lox/lox^ control mice, consumption of HF, but not HS, diet resulted in reduced time spent in the target quadrant during the probe trial, suggesting a hippocampal-dependent memory deficit. IR^lox/lox^;NPY^Cre/+^ mice had poor performance in the probe trial regardless of diet, suggesting a floor effect. This study did not find adverse effects of chronic sucrose intake on metabolic outcomes or hippocampal-dependent memory. These data also suggest that the effects of HF diet on hippocampal-dependent memory may be dependent on insulin signaling in hippocampal NPY cells.

## Introduction

Obesity has developed into a worldwide epidemic over the past 30 years, predominately due to adoption of a ‘Western’ diet in many countries. Western diets tend to include excessive consumption of calories derived from convenience foods (i.e. fast foods) high in refined sugar and saturated fat (Argueta and DiPatrizio, 2017). In addition to weight gain, Western diets can augment development of pathologies comorbid with obesity, including cardiovascular disease, insulin resistance and cognitive impairment (Jokinen, 2015; Alford et al., 2018). Obesity and obesity-related comorbidities cost the Australian economy billions of dollars per annum (The Obesity Collective, 2019). Additionally, a higher body mass index (BMI) in middle age is associated with lower cognitive performance in later life (Hassing et al., 2010). Therefore, the burdens of a Western diet will be endured for many more decades to come.

In rodent studies, high fat diets (HF) and high sugar diets (HS) have been linked to impaired performance in cognitive tasks such as the Morris Water Maze (MWM), which measures hippocampal-dependent spatial learning and memory (Vorhees and Williams, 2006; Kendig et al., 2013). The hippocampus has a high density of insulin receptors (IR), and mice consuming a HF diet for 12 weeks display systemic insulin resistance with a reduction in hippocampal IR gene expression (Liu et al., 2015). Hippocampal IRs are vital for glucose uptake within the hippocampus, as demonstrated by the finding that inserting anti-insulin antibodies into the rat hippocampus downregulates local glucose consumption. Importantly, this was correlated with impaired performance of spatial memory in the probe trial of the MWM (Wang et al., 2014). Similarly, mice that have a genetic knockout of hippocampal IR display peripheral glucose intolerance and impaired spatial memory (Soto et al., 2019) Notably though, previous work from our laboratory has demonstrated that intrahippocampal insulin infusion in obese mice can reverse HF-induced cognitive impairments displayed during MWM tasks (Gladding et al., 2018).

It is evident that insulin plays a key role in maintaining proper hippocampal functioning, including regulation of memory and learning tasks. Despite this knowledge, it remains unclear which neuronal mediators within the hippocampus are sensitive to insulin’s regulatory effects. Neuropeptide-Y (NPY) has dense expression within the hypothalamus and hippocampus, brain regions associated with food intake and spatial learning and memory, respectively (Luquet et al., 2005; Ghiasi et al., 2011; Loh et al., 2017). NPY is almost exclusively expressed from GABAergic neurons and has vital roles in neuronal excitability and synaptic transmission (Jinno and Kosaka, 2003; Marshall et al., 2017). Within the hippocampus, NPY has neuroprotective properties and can regulate cell proliferation (Duarte- Neves et al., 2016), while NPY injections into the dorsal hippocampus can increase memory retention in mice (Flood et al., 1989). Similarly, rats exposed to MWM tasks display upregulated NPY mRNA within the dentate gyrus of the hippocampus (Hadad-Ophir et al., 2014). Currently, there is limited research investigating the combined effects of diet and knockout of IRs on NPY expressing neurons and their possible contributions to hippocampal-dependent cognitive tasks. To address this, we utilized the Cre-lox recombination technique in mice to selectively knock out IRs on NPY neurons (IR^lox/lox^;NPY^Cre/+^). Mice were tested in the MWM after maintenance on dietary interventions, including HF, HS or a combination of HF and HS dietary treatment (HFHS). Based on these data, we hypothesize that mice with an NPY-specific IR knockout will display behavioral deficits during MWM performance, with consumption of HF and/or HS diets exacerbating these effects.

## Materials and Methods

### Animals

A conditional knockout mouse model was generated to selectively knockout the IR on NPY expressing neurons (IR^lox/lox^;NPY^Cre/+^). This mouse model has been validated in previous work, which functionally demonstrated that IRs were deleted from NPY neurons (Loh et al., 2017). To generate this conditional knockout, Floxed IR mice (IR^lox/lox^) (Brüning et al., 2000) were crossed with NPY^Cre/+^ mice (Qi et al., 2016) to generate double heterozygous mice IR^lox/+^;NPY^Cre/+^. These mice were then crossed again with IR^lox/lox^ mice to generate IR^lox/lox^;NPY^Cre/+^ mice. Breeding colonies were maintained by mating IR^lox/lox^ mice with IR^lox/lox^;NPY^Cre/+^. All mice were bred on a C57Bl/6J background.

Littermates that lacked the Cre recombinase enzyme (IR^lox/lox^) were used as controls as they express normal IR signaling within NPY-expressing neurons (Loh et al., 2017). This mouse line was maintained at Australian BioResources Ltd., Moss Vale, NSW, Australia, where genotyping was also performed. For behavioral studies, 48 male IR^lox/lox^;NPY^Cre/+^ and 48 male IR^lox/lox^ control mice were used. Mice were delivered to University of New South Wales at 8–12 weeks of age. Mice were housed two to four per cage (37 × 23 × 14 cm) under temperature-controlled conditions (22 ± 2°C) with a 12 h light-dark cycle (07:00 on–19:00 off). Upon arrival, mice were handled and allowed to become acclimated to their new environment for 1 week before dietary intervention began. For each genotype, mice were randomly assigned to one of four diets (control (CON), HF, HS or HFHS) for the reminder of the experiment; 12 weeks (12 mice in each dietary treatment group).

Body weights and food and sucrose intakes were measured using a manual averaging balance 3 times a week. Energy intake was calculated based on the quantity of food consumed and the known caloric density of the diets. Experimental procedures were approved by the University of New South Wales Animal Care and Ethics Committee (Ethics #16/21A) in accordance with the Australian Code of Practice and Use of Animals for Scientific Purposes.

### Diets

The control diet contained 7% fat w/w (15.9% calories from fat) and the high fat (HF) diet contained 21% fat w/w (40.6% calories from fat). Apart from fat and carbohydrate content, diets had identical compositions (see Supplementary Table S1), where a portion of the fat (ghee) was replaced by carbohydrate (wheat starch) in the CON diet. Diets were based on the American Institute of Nutrition Guidelines (AIN93) and were therefore nutritionally complete. Half of the animals on each diet received 10% weight per volume (w/v) sucrose solution. 10% sucrose solution was used because its caloric density (around 0.4 kcal/g) is similar to that found in sugar sweetened beverages on the market (Kendig et al., 2013). The sucrose solution consisted of ≥99.5% Sucrose (Sigma-Aldrich Co. LLC, Castle Hill, NSW, Australia) dissolved in tap water. All animals had ad libitum access to tap water. Diets were replenished every few days.

### Morris Water Maze

The protocol used for training and testing in the MWM was based on established methods (Vorhees and Williams, 2006; Gladding et al., 2018). The MWM was conducted during the light cycle for all cohorts of animals in a round tank (120 cm diameter × 60 cm depth) that was filled with 30 cm of water. Experimenters left the room during testing of animals. Mice were trained to use distal spatial cues surrounding the maze to locate a hidden escape platform situated beneath the surface of the water. The water was kept at 22°C and rendered opaque by the addition of a non-toxic tempera powder. On day 1: The escape platform was positioned in the center of the pool. Familiarization began by placing the mouse on the submerged platform for 30 s. The mouse was removed from the platform and released from the pool edge with its head facing the platform. The mouse was guided towards the platform if it did not reach the platform in 60 s, or was placed onto the platform after 90 s for 10 s. This procedure was repeated for two trials with a 5 min inter-trial period. Days 2–4: The escape platform was positioned at the center of the NW quadrant. Each mouse received four trials per day over three consecutive days with an inter-trial interval of 5 min. Each trial involved the release of the mouse from one of four fixed points (N, S, E, W). The starting positions were assigned in random order, to prevent the use of a praxis strategy (using a learned sequence of movements), rather than a spatial mapping strategy. Data from the four daily trials were averaged each day. Mice were dried and warmed after each training trial. On Day 5, the 90 s probe trial was performed, where the platform was removed. The time spent in the target quadrant and path length in the probe trial were scored using EthoVision (Noldus Information Technology, XT v5.1, Netherlands).

### Glucose Tolerance Test (GTT)

The GTT was conducted in mice following the completion of behavioral testing. Following a 4-h fast, the tip of the tail was cut (∼1 mm) and baseline glucose measured (∼5 uL) (Accu-Chek; Roche Diagnostics, IN) and 50 uL blood collected for measurement of blood insulin and leptin levels by ELISA (Crystal Chem, IL). Mice were then injected intraperitoneally with a glucose solution (∼200 μL/mouse; 1 g/kg). Blood glucose was assessed again at 15-, 30-, 60-, and 120-min post injection, and blood was collected for insulin measurement once more at 15 min.

### Statistical Analysis

The study employed a 2 × 2 × 2 between subjects design with genotype (IR^lox/lox^, IR^lox/lox^;NPY^Cre/+^), sucrose water (HS, CON) and high fat diet (HF, CON) as the factors. Data were analyzed using Statistica 12.0 (Dell Software, NSW, Australia) and is presented as means with standard errors. Data were first tested for normality and a factorial ANOVA was conducted for energy intake, plasma leptin, area under the curve (AUC) and probe trial. A mixed design ANOVA, within (time) and between (genotype, HS, HF), was conducted for body weight, blood glucose, plasma insulin, mean escape latencies) followed by Tukey’s honest significance difference (HSD) test for post-hoc analysis when a significant interaction effect was observed. Differences were accepted as statistically significant at *p* < 0.05.

## Results

### Body weight, Energy Intake, Plasma Leptin

**FIGURE 1:**
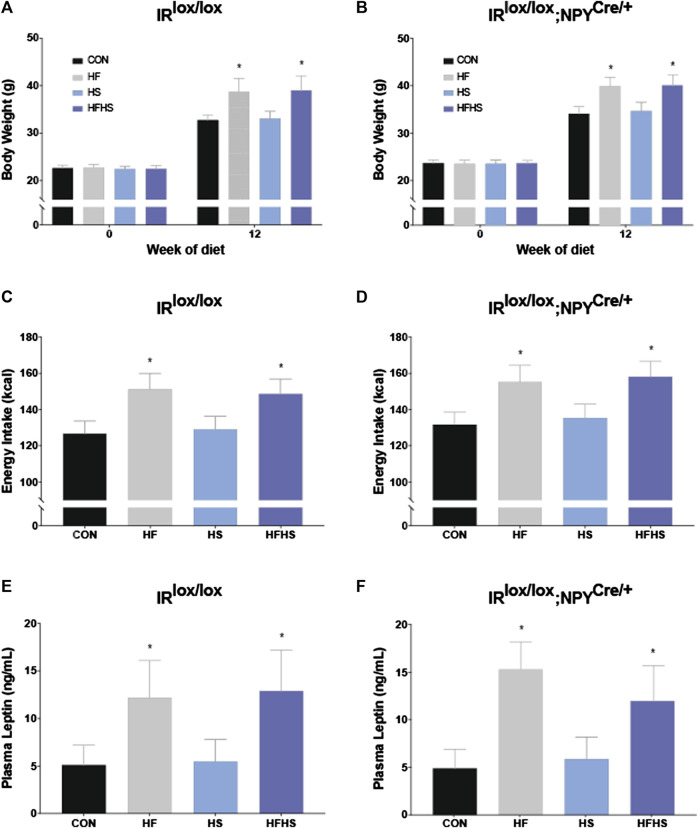
*Body weight, energy intake and plasma leptin.* Body weights of IR^lox/lox^ and IR^lox/lox^;NPY^Cre/+^ mice increased after 12 weeks of diet intervention in all treatment groups. Body weight was further upregulated after 12 weeks of diet in HF and HFHS groups compared to CON and HS diet group **(A, B)**. Energy intake of IR^lox/lox^ and IR^lox/lox^;NPY^Cre/+^ mice was higher in HF and HFHS treatment groups compared to CON and HS diet group **(C, D)**. Plasma leptin recordings of IR^lox/lox^ and IR^lox/lox^;NPY^Cre/+^ mice were higher in HF and HFHS treatment groups compared to CON and HS group. **(E, F)**. CON = chow diet, HF = high fat diet, HS = high sugar diet, HFHS = high fat and high sugar diet. Values are expressed as mean ± SEM. * = p < 0.05. Analysed by a mixed ANOVA for body weight and a factorial ANOVA for energy intake and plasma leptin. IR^lox/lox^; *n* = 48 (12/diet group). IR^lox/lox^;NPY^Cre/+^; *n* = 48 (12/diet group).

Across all dietary treatment groups, both IR^lox/lox^ and IR^lox/lox^;NPY^Cre/+^ mice displayed significantly increased body weight after 12 weeks of diet intervention. This weight gain was significantly greater in mice that received HF and HFHS diet. There was no significant effect of genotype on body weight at either baseline or 12 weeks time points (Figures 1A, B; *p* < 0.05). There was a main effect of HF and HFHS on total energy intake, with HF and HFHS fed IR^lox/lox^ and IR^lox/lox^;NPY^Cre/+^ mice consuming more than CON and HS mice, with no differences between genotypes (Figures 1C, D; *p* < 0.05). Plasma leptin levels reflected the same trend as the body weight and intake data, with HF and HFHS fed animals displaying significantly higher plasma leptin levels than CON and HS animals, regardless of genotype (Figures 1E, F; *p* < 0.05).

### Glucose Tolerance Test (GTT)

**FIGURE 2:**
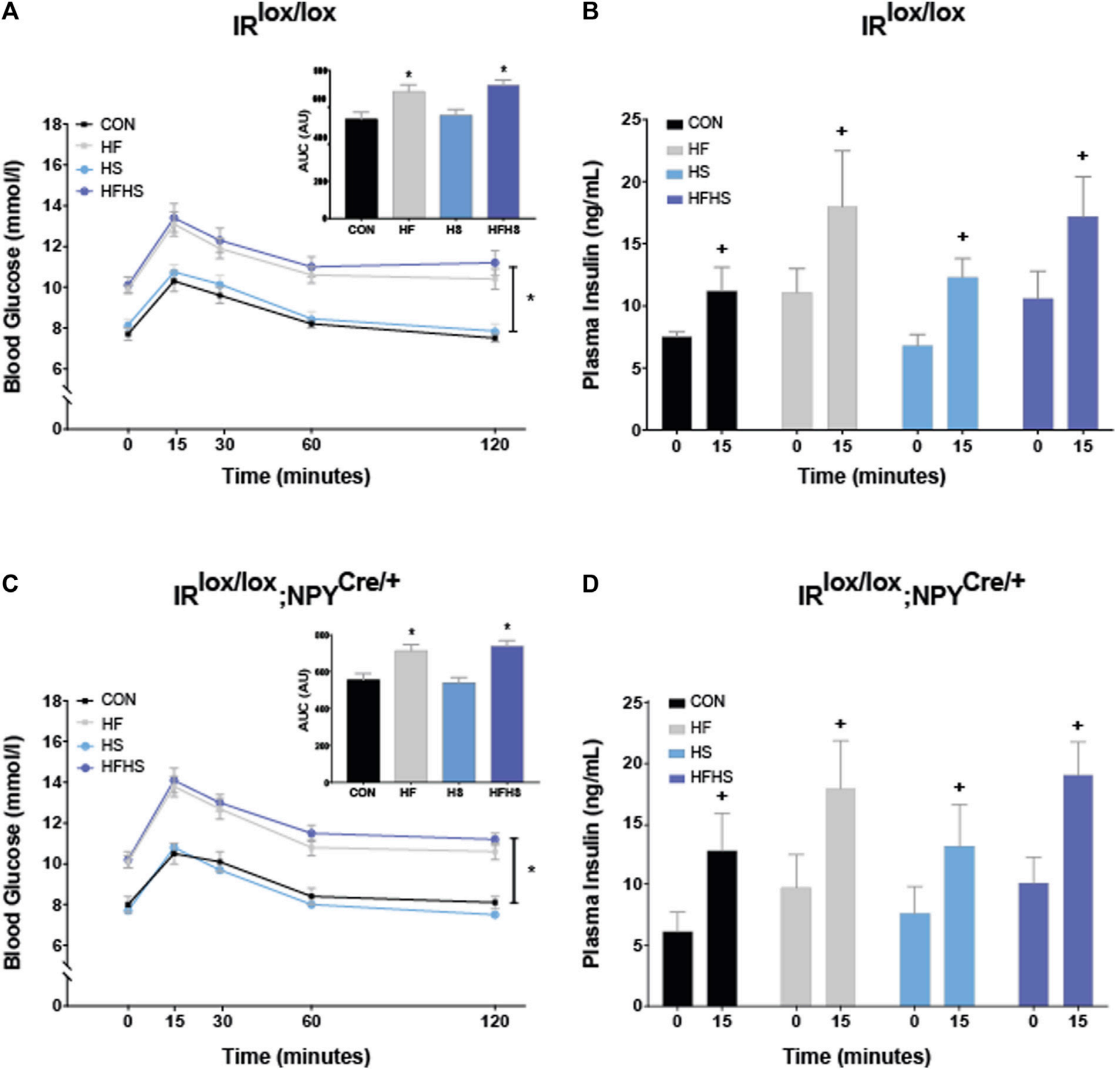
*Blood glucose and insulin.* Blood glucose levels in IR^lox/lox^ control mice in response to peripheral glucose injection over 120 min. HF and HFHS treatment groups displayed reduced glucose tolerance in both IR^lox/lox^ and IR^lox/lox^;NPY^Cre/+^ mice compared to CON and HS groups. Insets represent arbitrary units (AU) for area under the curve (AUC) values in IR^lox/lox^ and IR^lox/lox^;NPY^Cre/+^ mice. AUC results show a main effect of diet, with HF and HFHS treatment groups displaying increased AUC following peripheral glucose injection compared to CON and HS diet groups **(A, C)**. There was a within-subjects effect of time on insulin sensitivity within all dietary treatment groups in both IR^lox/lox^ and IR^lox/lox^;NPY^Cre/+^ mice **(B, D)**. CON = chow diet, HF = high fat diet, HS = high sugar diet, HFHS = high fat and high sugar diet. Values are expressed as mean ± SEM. + = main effect of time, * = p < 0.05. Analysed by a mixedANOVA **(A–D)** and factorial ANOVA (insets of A, C) followed by Tukey’s honest significance difference (HSD) test. IR^lox/lox^; *n* = 48 (12/diet group). IR^lox/lox^;NPY^Cre/+^; *n* = 48 (12/diet group).

To examine differences in peripheral glucose metabolism, glucose tolerance was examined in IR^lox/lox^ and IR^lox/lox^;NPY^Cre/+^ mice. There was a main effect of diet, with animals on HF and HFHS diet displaying increased blood glucose at all time points compared to CON and HS animals, regardless of genotype. All animals displayed a similar increase in blood glucose 15 min after peripheral glucose injection. The area under the curve (AUC) was calculated for blood glucose values following peripheral glucose injection. There was a main effect of diet, with mice on HF and HFHS diet showing increased AUC arbitrary values compared to CON and HS. There was no effect of genotype (Figures 2A, C; *p* < 0.05). There was a significant within-subjects effect observed for plasma insulin levels, with all animals displaying increased plasma insulin levels 15 min following glucose injection. There were no significant effects of genotype or dietary interventions on baseline insulin or insulin release following glucose injection (Figures 2B, D; *p* < 0.05).

### Morris Water Maze

**FIGURE 3:**
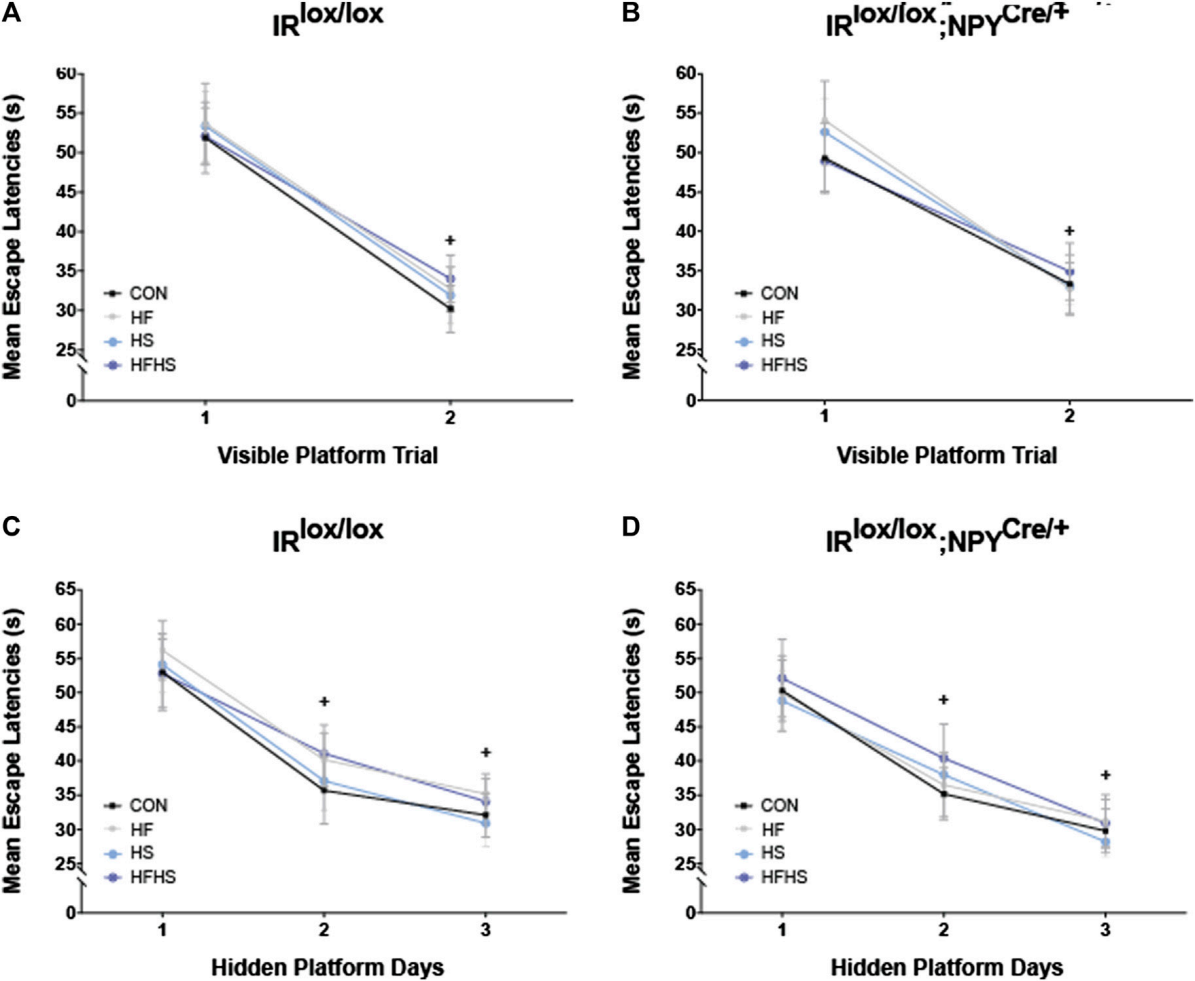
*Morris Water Maze performance–Mean Escape Latencies.* Mean escape latencies were collected for each trial day to assess performance over time. Across all dietary treatment groups, mean escape latencies were significantly decreased on Day 2 of visible platform trial for IR^lox/lox^ control mice and IR^lox/lox^;NPY^Cre/+^ mice **(A, B)**. IR^lox/lox^ mice and IR^lox/lox^;NPY^Cre/+^ mice reached the platform faster across the hidden platform days in all treatment groups **(C, D)**. CON = chow diet, HF = high fat diet, HS = high sugar diet, HFHS = high fat and high sugar diet. Values are expressed as mean ± SEM. + = main effect of time, *p* < 0.05. Analysed by a mixed ANOVA followed by Tukey’s honest significance difference (HSD) test. IR^lox/lox^; *n* = 48 (12/diet group). IR^lox/lox^;NPY^Cre/+^; *n* = 48 (12/diet group).

Both IR^lox/lox^ and IR^lox/lox^;NPY^Cre/+^ mice demonstrated similar escape latencies on Day 1 and displayed improvements across visible platform trials regardless of dietary intervention (Figures 3A, B; *p* < 0.05). Similarly, both genotypes and all dietary groups reached the platform faster over the hidden platform training days (Figures 3C, D; *p* < 0.05).

**FIGURE 4:**
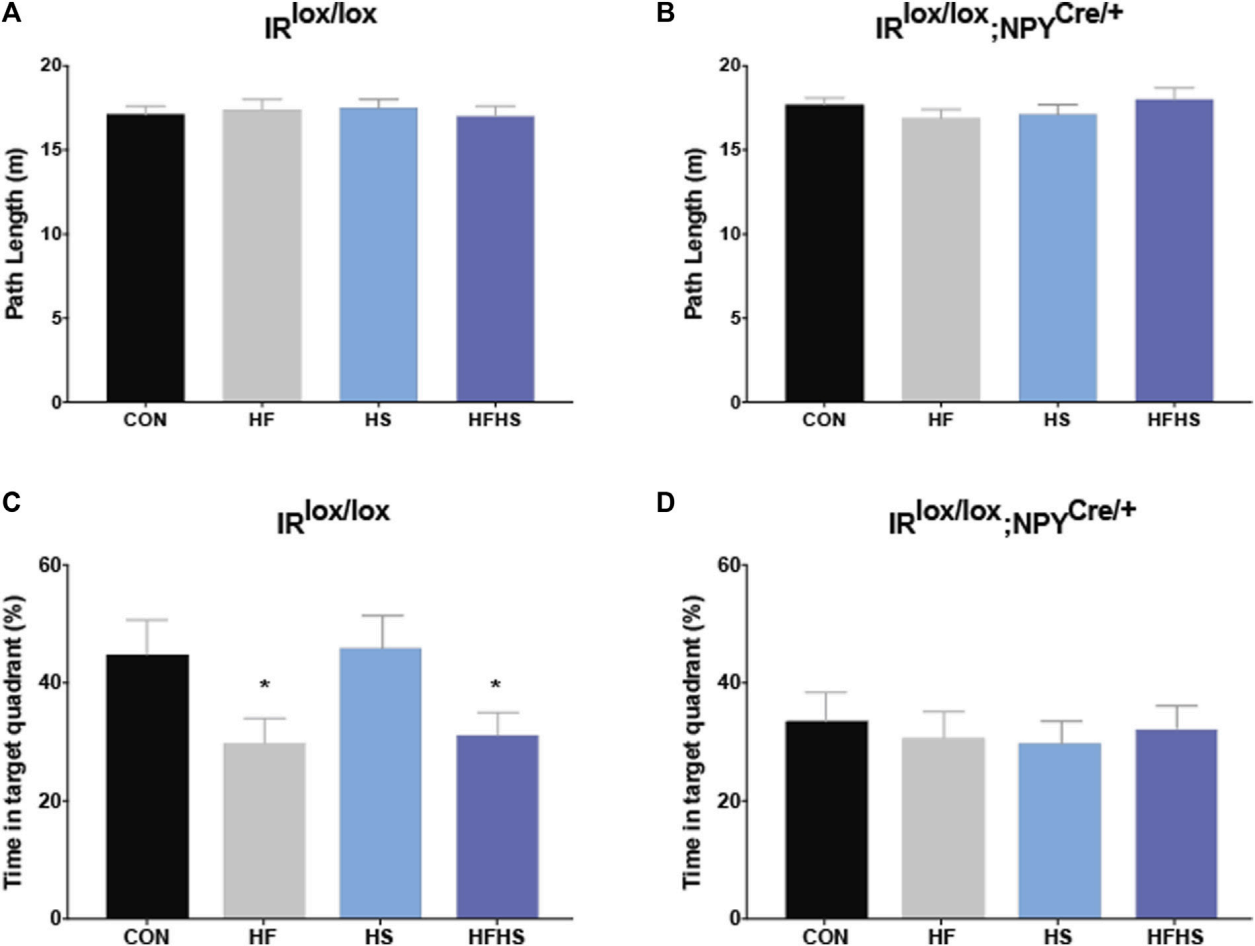
*Morris Water Maze Performance - Probe trial.* Time spent in the target quadrant was measured to assess hippocampal-dependent memory. IR^lox/lox^ control mice and IR^lox/lox^;NPY^Cre/+^ mice across all dietary groups swam similar path lengths in the target quadrant during the probe trial **(A, B)**. IR^lox/lox^ mice fed a HF or HFHS diet spent less time in the target quadrant relative to IR^lox/lox^ mice fed a CON or HS diet **(C)**. There was a main effect of genotype on time spent in the target quadrant, with IR^lox/lox^;NPY^Cre/+^ mice in all dietary treatment groups spending less time in the target quadrant than IR^lox/lox^ CON mice **(D)**. CON = chow diet, HF = high fat diet, HS = high sugar diet, HFHS = high fat and high sugar diet. Values are expressed as mean ± SEM. * = p < 0.05. Analysed by a factorial ANOVA. IR^lox/lox^; *n* = 48 (12/diet group). IR^lox/lox^;NPY^Cre/+^; *n* = 48 (12/diet group).

During the probe test, all groups swam similar path lengths (Figures 4A, B). There was a main effect of diet for IR^lox/lox^ mice, with HF and HFHS fed mice spending less time in the target quadrant compared with CON and HS fed mice (Figure 4C; *p* < 0.05). There was a main effect of genotype on the time spent in the target quadrant, with IR^lox/lox^;NPY^Cre/+^ mice in all dietary treatment groups spending reduced time in the target quadrant compared to CON and HS IR^lox/lox^ mice (Figures 4C, D; *p* < 0.05).

## Discussion

Long-term consumption of foods high in fat and sugar can lead to reduced cognitive performance (Fu et al., 2017; Gladding et al., 2018), yet the specific neuronal populations underlying these effects remains unclear. This study aimed to examine whether *ad libitum* consumption of HS, HF, or HFHS diets impaired hippocampal-dependent spatial memory performance in the MWM, and, if so, whether the deficit was potentiated by impaired insulin signaling in NPY neurons. Specific deletion of IRs on NPY neurons was achieved using a conditional IR^lox/lox^;NPY^Cre/+^ knockout mouse model. This mouse model has been previously validated using phosphorylated-Akt (p-Akt) as a measure of insulin signaling in NPY neurons (Manning and Cantley, 2007; Loh et al., 2017). Intracerebroventricular infusion of insulin was administered to mice which caused downstream activation of p-Akt in the brain of IR^lox/lox^ mice but not IR^lox/lox^;NPY^Cre/+^ mice (Loh et al., 2017). Additionally, immunohistochemical labeling of p-Akt reveals a substantial downregulation of insulin signaling within the hippocampus of IR^lox/lox^;NPY^Cre/+^ mice (Goodman et al., 2022).

Mice fed a HF or HFHS diet gained more weight relative to HS or chow diets over the course of the experiment. The exposure of diets extended for 12 weeks and were administered during adulthood. This dietary duration is consistent with other studies examining impacts of long-term high-fat and/or chronic sucrose interventions on rodent physiology (Aoi et al., 2011; Chang et al., 2021; Odom et al., 2021). It was interesting, although not altogether unexpected, that the HS diet did not cause significant weight gain. It has previously been reported that mice fed a HF diet display increased obesity and insulin resistance compared to mice fed a HS diet (Perazza et al., 2020). Additionally, HF diets can cause significant alterations to the size and morphology of adipocytes as well as increasing inflammatory mediators in visceral adipose tissue (Dobner et al., 2017). Conversely, HS diets can produce less overt effects on mediators of adiposity in mice (Chen et al., 2011; Tang et al., 2014; Dobner et al., 2017). In the current study, plasma leptin levels were also upregulated in the HF and HFHS treatment groups, which reflects blunted leptin sensitivity and therefore a desensitization to satiety signaling within the brain (Ryan et al., 2012). This could encourage overeating and exacerbate increased caloric intake of HF and HFHS fed mice.

Although HF and HFHS mice displayed decreased glucose tolerance during the GTT, there was no effect of diet on peripheral insulin sensitivity. A lack of dietary interaction was not unexpected, as previous data from our laboratory has demonstrated that a HF diet does not significantly affect plasma insulin levels following an intraperitoneal glucose injection (1 g/kg of body weight) in mice (Gladding et al., 2018). Future studies could employ an oral glucose tolerance test, as oral glucose administration is associated with greater changes in blood insulin levels than intraperitoneal administration (Small et al., 2022). Indeed, results from oral glucose tolerance tests have previously demonstrated greater elevations in plasma insulin in HF vs. chow-fed mice 15 min following administration (Andrikopoulos et al., 2008).

There was no effect of genotype on blood glucose (including AUC values) and plasma insulin levels following peripheral glucose injection. These results could be considered surprising due to the conditional IR knockout, however, the current data aligns with previous GTT results using this IR^lox/lox^;NPY^Cre/+^ mouse model (Goodman et al., 2022). To specify, knocking out IRs on NPY neurons does not elicit any obvious effects on peripheral insulin sensitivity and glucose tolerance. This could be evidence of a previously unknown regulation between central and peripheral insulin regulation.

Moreover, there were no significant differences between IR^lox/lox^ and IR^lox/lox^;NPY^Cre/+^ mice with respect to body weight, energy intake and plasma leptin levels. Loh et al. (Loh et al., 2017) demonstrated that male IR^lox/lox^;NPY^Cre/+^ mice have upregulated basal daily energy intake and increased fat mass compared to controls, however only when measured at 16 weeks of age. Additionally, there were body weight differences observed at 4–6 weeks of age, with this effect plateauing with increased age (Loh et al., 2017). Results from a recent study of ours (Goodman et al., 2022) found that 6 month old IR^lox/lox^;NPY^Cre/+^ mice, but not 12 or 24 month old mice, had increased body weight compared to controls. From these data, it appears that knocking out IR on NPY neurons causes alterations in energy balance, but only during specific stages of the life cycle. It is unclear why no body weight differences were observed within the current experimental paradigms, however it may reflect a complex interaction between central insulin signaling and energy balance.

In the MWM, neither knockout of IRs on NPY neurons nor dietary manipulations affected the animals’ capability to ascertain the platform’s location. There were no significant differences between any groups in the escape latencies across the hidden platform training trials. All groups swam similar path lengths in the probe trial, and hence between-group differences could not be attributed to motivational or sensorimotor deficits. Interestingly, during the probe trial, IR^lox/lox^;NPY^Cre/+^ mice spent less time in the target quadrant than IR^lox/lox^ mice, indicating that loss of IRs on NPY cells leads to a hippocampal-dependent spatial memory deficit. This poor performance in the probe trial was displayed in all diet intervention groups, including the control diet group, of IR^lox/lox^;NPY^Cre/+^ mice, suggesting a floor effect. These data suggest that the neuronal mediators responsible for maintaining hippocampal-dependent spatial memory are IRs expressed on NPY neurons. Importantly, behavior displayed from the MWM is well established to be hippocampal-dependent. Therefore, despite expression of NPY within other regions of the brain, such as the hypothalamus, the deficits observed in the current study appear to be predominately mediated by NPY-located hippocampal neurons. This is supported by immunohistochemical validation experiments from a recent publication of ours, which demonstrated that insulin signaling within the dentate gyrus of the hippocampus is significantly downregulated in IR^lox/lox^;NPY^Cre/+^ mice (Goodman et al., 2022). Although these results do suggest that insulin signaling on NPY neurons plays a key role in spatial memory, they do not rule out the possibility that additional neuronal subtypes are involved in potentiating hippocampal cognitive functioning.

Cognitive and spatial memory deficits are known to be exacerbated by lifestyle and dietary habits (Spencer et al., 2017), therefore we sought to determine how a HF, HS and HFHS diet affected MWM performance. IR^lox/lox^ control mice on HF or HFHS diets displayed reduced time in the target quadrant, consistent with the previously observed effects of HF and HFHS diets on spatial learning and memory (Gergerlioglu et al., 2016; Takechi et al., 2017; Park et al., 2018). Interestingly, these results were similar to the poor MWM performance displayed by IR^lox/lox^;NPY^Cre/+^ mice during the probe trial. The mechanisms by which HF and HFHS diets reduce MWM performance in IR^lox/lox^ mice remain unclear, however a HF diet can lead to reduced hippocampal intrinsic excitability (Underwood and Thompson, 2016) and downregulation of hippocampal insulin signaling (Petrov et al., 2015; Fu et al., 2017). Impaired insulin signaling in the hippocampus has been associated with spatial learning and memory impairments (Wang et al., 2014), likely due to a downregulation of synaptic transmission (Grillo et al., 2015). Moreover, a HF diet can reduce insulin-dependent microvascular regulation in the hippocampus, which is associated with reduced performance on spatial and learning tasks in rats (Fu et al., 2017).

Importantly, the performance of IR^lox/lox^ mice during the MWM probe trial also suggests that a HS diet does not significantly affect hippocampal-dependent spatial memory. While some previous work has found that long-term sucrose consumption leads to reduced MWM performance (only when administered in the juvenile (Kendig et al., 2013) and adolescent (Hsu et al., 2015) periods of development in rodents) and impaired place recognition in memory tasks (Beilharz et al., 2014), other reports demonstrate that rats and mice fed a HS diet do not display impaired spatial memory, whereas animals fed a HF diet do (Pyndt Jørgensen et al., 2014; Gergerlioglu et al., 2016). Accordingly, in the current study, there were no differences in probe trial performance between HF and HFHS diet mice, presumably due to the relatively neutral effect a HS diet appears to have on the metabolism and behavior of the current cohort of mice. Additionally, the current mice were exposed to dietary intervention during adulthood, whereas previous significant findings of chronic sucrose exposure on MWM performance were in young mice (Kendig et al., 2013; Hsu et al., 2015) This implies a distinct effect of age on susceptibility to sucrose-dependent cognitive dysfunction.

It could be argued that poor probe trial performance displayed from HF and HFHS animals could be a direct consequence of an obese phenotype. However, cognitive deficits are often a result of factors related to diet but independent of adiposity (Beilharz et al., 2015; Vinuesa et al., 2016), such as neuroinflammation (Valdearcos et al., 2014; Duffy et al., 2019). In fact, in both human (Holloway et al., 2011) and rodent (Kanoski and Davidson, 2010) studies, cognitive deficits can appear only 1 week after exposure to a high energy diet, before any significant weight changes can occur. Therefore, it is likely that the cognitive deficits observed in the current study were a result of macronutrient content as opposed to obesity.

In conclusion, the results of the current experiment demonstrate that IR^lox/lox^ mice fed a HF and HFHS diet exhibit impaired performance in the probe trial of the MWM compared with mice maintained on a chow diet. Animals with IR knockout on NPY neurons also display poor performance on the probe trial, independent of dietary intervention. Through the use of our conditional knockout mouse model, we provide tentative evidence that hippocampal-dependent spatial memory may be mediated by IRs NPY-expressing cells. Interestingly, mice fed a HS diet did not display cognitive deficits in the MWM. This suggests that specific macronutrients can differentially affect neuronal activity and spatial memory. Collectively, these findings support our understanding of how IR signaling on distinct neuronal populations affects cognition, which could aid in developing therapeutics to alleviate pathologies associated with insulin resistance, such as obesity and type-2 diabetes mellitus.

## Data Availability

The original contributions presented in the study are included in the article/Supplementary Material, further inquiries can be directed to the corresponding author.
