# Supplementary material for: The Effect of Dietary Fat and Sucrose on Cognitive Functioning in Mice Lacking Insulin Signaling in Neuropeptide Y Neurons
Source: Front Physiol. 2022 Apr 26;13:841935. doi: 10.3389/fphys.2022.841935 (PMC9086626; doi:10.3389/fphys.2022.841935)
Supplement: Supplementary file 1 [file DataSheet1.PDF]

| | Ingredients (g/100g) | |
|---|---|---|
| | NFD | HFD |
| Carbohydrates | | |
| Sucrose | 34.15 | 34.15 |
| Cellulose | 5.00 | 5.00 |
| Wheat Starch | 27.20 | 13.20 |
| Dextrinised Starch | 2.24 | 2.24 |
| Fat | | |
| Safflower Oil | 1.50 | 1.50 |
| Clarified Butter (Ghee) | 5.50 | 19.50 |
| Protein | | |
| Casein Acid | 19.50 | 19.50 |
| Trace Minerals | .14 | .14 |
| Dl Methionine | .30 | .30 |
| Lime | 1.71 | 1.71 |
| Salt | .26 | .26 |
| Potassium DiHydrogen Phosphate | .69 | .69 |
| Potassium Sulphate | .16 | .16 |
| Potassium Citrate | .25 | .25 |
| Choline Chloride 75% w/w | .25 | .25 |
| USP Cholesterol | .15 | .15 |
| OXICAP E2 (66%) | Trace | Trace |
